# Supplementary figures and images for: Nutritional Immunity, Zinc Sufficiency, and COVID-19 Mortality in Socially Similar European Populations
Source: Front Immunol. 2021 Sep 17;12:699389. doi: 10.3389/fimmu.2021.699389 (PMC8484327; doi:10.3389/fimmu.2021.699389)

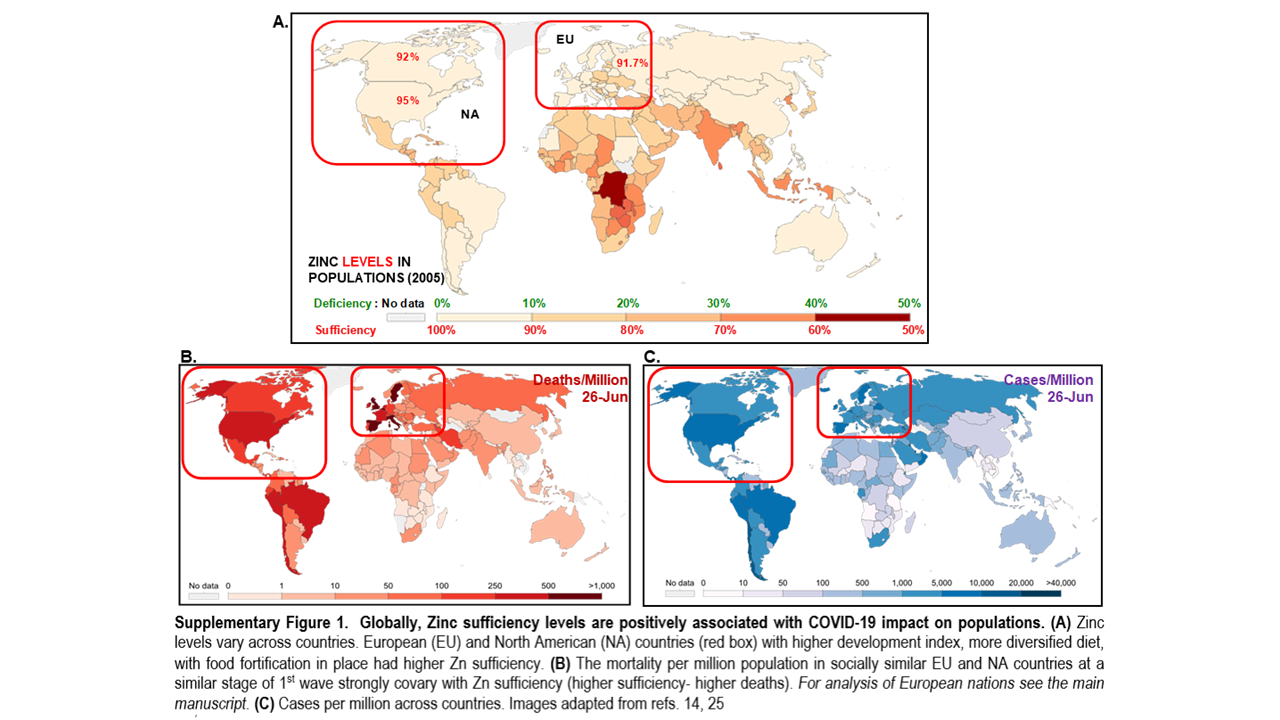

Supplement: Supplementary file 2 [file Image_1.png]
