# Supplementary material for: Nutritional Immunity, Zinc Sufficiency, and COVID-19 Mortality in Socially Similar European Populations
Source: Front Immunol. 2021 Sep 17;12:699389. doi: 10.3389/fimmu.2021.699389 (PMC8484327; doi:10.3389/fimmu.2021.699389)
Supplement: Supplementary file 3 [file Image_2.pdf]

A.

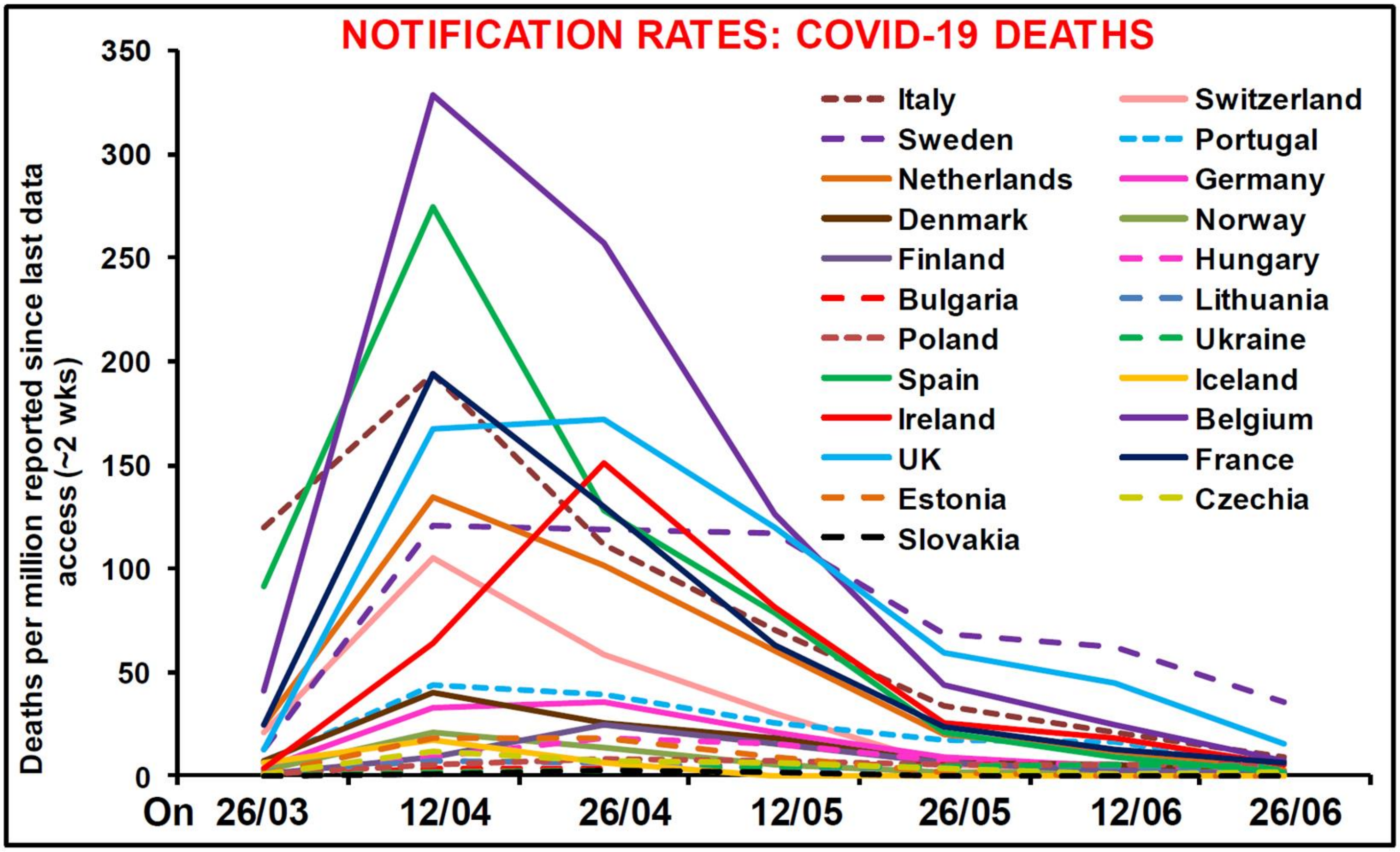

B.

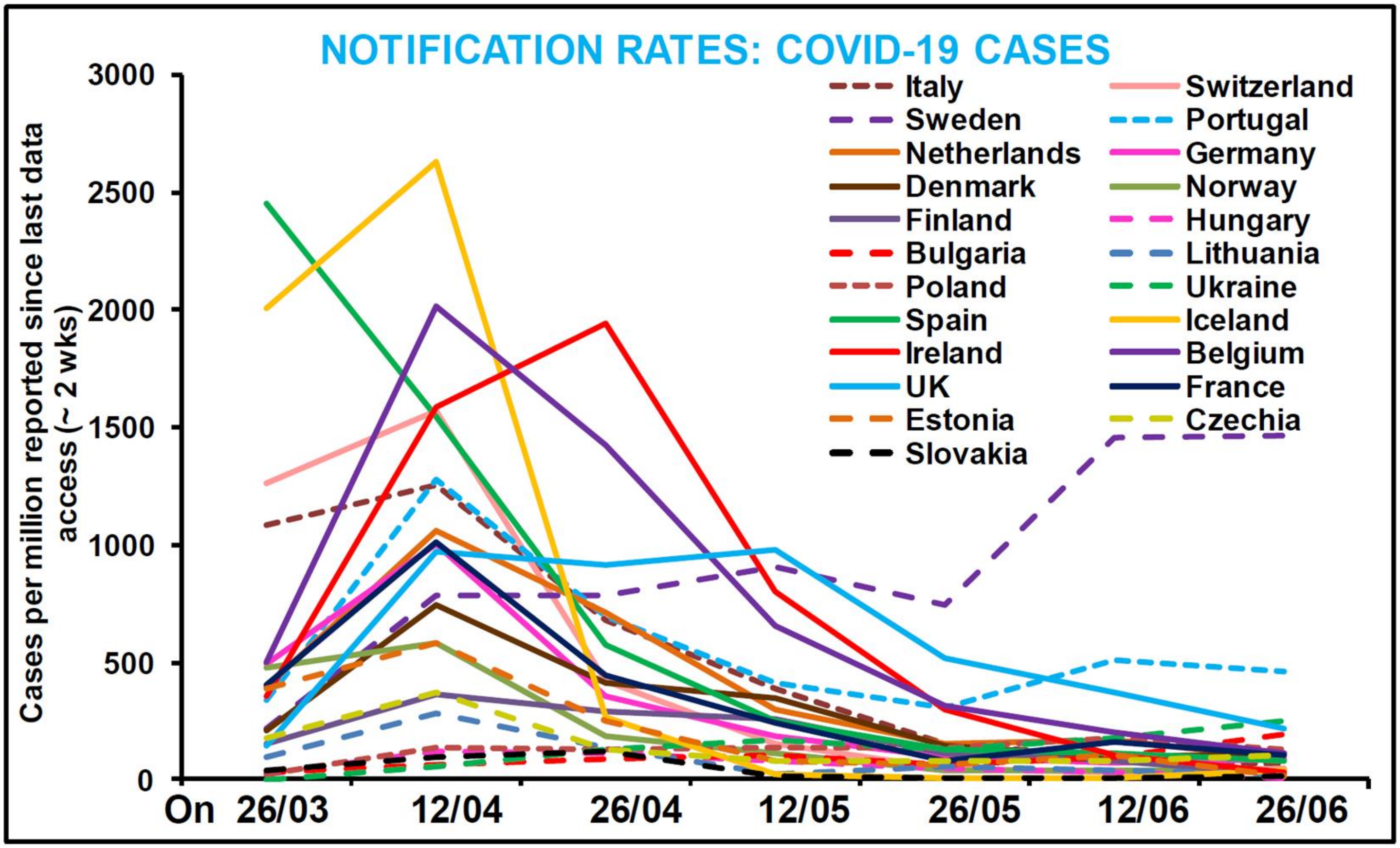

Supplementary Figure 2. The wave of COVID-19 deaths (8 out of 10 were elderly) **(A)** and incidences (majority younger – without dysregulated Zn homeostasis) **(B)** as it traversed the countries analyzed in the current study
